# Improper preanalytical processes on peripheral blood compromise RNA quality and skew the transcriptional readouts of mRNA and LncRNA

**DOI:** 10.3389/fgene.2022.1091685

**Published:** 2023-01-04

**Authors:** Yinli He, Lele Dong, Hongyang Yi, Linpei Zhang, Xue Shi, Lin Su, Baoyu Gan, Ruirui Guo, Yawen Wang, Qinying Luo, Xiaojiao Li

**Affiliations:** ^1^ BioBank, The First Affiliated Hospital of Xi’an Jiaotong University, Xi’an, Shaanxi, China; ^2^ Department of Pharmacy, The First Affiliated Hospital of Xi’an Jiaotong University, Xi’an, Shaanxi, China; ^3^ National Clinical Research Centre for Infectious Diseases, The Third People’s Hospital of Shenzhen, The Second Affiliated Hospital of Southern University of Science and Technology, Shenzhen, Guangdong, China

**Keywords:** peripheral blood leukocytes (PBLs), preanalytical processes, RNA quality, transcriptional readouts, COPD, TNBC

## Abstract

Genetic and epigenetic reprogramming caused by disease states in other tissues is always systemically reflected in peripheral blood leukocytes (PBLs). Accurate transcriptional readouts of Messenger RNA (mRNA) and Long non-coding RNA (lncRNA) in peripheral blood leukocytes are fundamental for disease-related study, diagnosis and treatment. However, little is known about the impact of preanalytical variables on RNA quality and downstream messenger RNA and Long non-coding RNA readouts. In this study, we explored the impact of RNA extraction kits and timing of blood placement on peripheral blood leukocyte-derived RNA quality. A novel enhanced evaluation system including RNA yields, purity, RNA integrity number (RIN) values and β-actin copies was employed to more sensitively identify RNA quality differences. The expression levels of informative mRNAs and Long non-coding RNAs in patients with chronic obstructive pulmonary disease (COPD) or triple-negative breast cancer (TNBC) were measured by Quantitative reverse transcription polymerase chain reaction (qRT–PCR) to investigate the impact of RNA quality on transcriptional readouts. Our results showed that the quality of RNA extracted by different kits varies greatly, and commercial kits should be evaluated and managed before batch RNA extraction. In addition, the quality of extracted RNA was highly correlated with the timing of blood placement, and the copy number of β-actin was significantly decreased after leaving blood at RT over 12 h. More importantly, compromised RNA leads to skewed transcriptional readouts of informative mRNAs and Long non-coding RNAs in patients with chronic obstructive pulmonary disease or triple-negative breast cancer. These findings have significant implications for peripheral blood leukocyte-derived RNA quality management and suggest that quality control is necessary prior to the analysis of patient messenger RNA and Long non-coding RNA expression.

## 1 Introduction

Peripheral blood leukocytes (PBLs) often act as proxies for disease states since they can systemically reflect genetic and epigenetic reprogramming in other tissues (Glade et al., 1968; Vorup-Jensen et al., 2005; Delobel et al., 2010; Javierre et al., 2010; Kiltschewskij and Cairns, 2020; Valero et al., 2020; Marczyk et al., 2021). RNA, which is the most important fraction of leukocytes, plays a pivotal role in understanding the transcriptomic profile of diseases (Raeymaekers, 1993). Messenger RNA (mRNA) is a pivotal molecule of life and is involved in almost all aspects of cell biology (Sahin et al., 2014). Long noncoding RNAs (lncRNAs) are a class of RNA transcripts with a length greater than 200 nucleotides that exert their functions by regulating gene expression and functions at transcriptional, translational, and posttranslational levels (Chi et al., 2019; Volovat et al., 2020). Any changes in mRNA and lncRNA expression levels may lead to inflammation and malignant disease, and aberrantly expressed mRNAs and lncRNAs are considered strong biomarkers in diseases. Hence, accurate transcriptional readouts of informative mRNAs and lncRNAs are fundamental for disease-related studies, diagnosis and treatment. RNA is frangible and prone to degradation (Auer et al., 2003; Zychowska et al., 2020), and the process of RNA extraction is susceptible to the variability of many factors, such as blood storage temperature and collection tubes (Delobel et al., 2010; Tang et al., 2019) (De Cecco et al., 2009; Rudloff et al., 2010; Hatzis et al., 2011). However, little is known about the impact of RNA extraction kits and timing of blood placement on RNA quality and downstream mRNA and lncRNA analysis.

RNA yields, purity and RNA integrity number (RIN) are routinely used as standard indicators to estimate RNA quality (Imbeaud et al., 2005; Schroeder et al., 2006). Recently, some studies suggested that these conventional indicators were not sensitive enough to discern between low- and high-quality RNA (Ribeiro-Silva et al., 2007; Greytak et al., 2015; Webster et al., 2015; Hester et al., 2016), implying that a novel evaluation system should be constructed to more accurately identify RNA quality inconsistencies. Fragmented RNA always leads to lower effective RNA inputs and further compromises the copy number of housekeeping genes in cDNA, which can be sensitively detected by digital droplet PCR (ddPCR) (Hindson et al., 2011; Pinheiro et al., 2012; Millier et al., 2017; Poh et al., 2020). Therefore, housekeeping gene copies may provide an enhanced indicator for RNA quality evaluation.

Quantitative reverse transcription polymerase chain reaction (qRT–PCR) is the most commonly used technique for detecting mRNA and lncRNA expression and verifying the candidates of RNA-seq (Nolan et al., 2006). Some reports have demonstrated that the cycle threshold (Ct) value had an opposite trend compared to the RIN (Wang et al., 2015), and RNA integrity significantly affected the 18 s, 28 s and IL-1β crossover point values (Fleige and Pfaffl, 2006). However, little is known about the impact of RNA quality on ΔΔCt values, which is the most commonly used indicator for representing the relative expression alteration in mRNA and lncRNA (Livak and Schmittgen, 2001). In this study, we first explored the impact of different RNA extraction kits and the timing of blood placement on the quality of PBL-derived RNA. A novel evaluation system including RNA yields, purity, RIN values and β-actin copies was employed to identify RNA quality inconsistencies. The expression levels of informative mRNAs and lncRNAs in patients with chronic obstructive pulmonary disease (COPD) or triple-negative breast cancer (TNBC) were detected by qRT–PCR to investigate the impact of RNA quality on mRNA and lncRNA readouts. Our results found that the quality of PBL-derived RNA extracted by different commercial kits varies greatly, suggesting that kit evaluation and management should be performed before batch RNA extraction. Additionally, the timing of blood placement should be limited to 12 h to obtain high-quality RNA. More importantly, the relative transcriptional readouts of the aberrantly expressed mRNAs and LncRNAs in patients with COPD or TNBC were heavily dependent on the quality of extracted RNA, and improper preanalytical processes resulted in skewed qRT–PCR results. This study thus comprehensively evaluated the preanalytical processes on PBL-derived RNA quality and downstream mRNA and lncRNA readouts.

## 2 Results

### 2.1 The quality of PBL-derived RNA extracted by different commercial kits varies greatly

**FIGURE 1:**
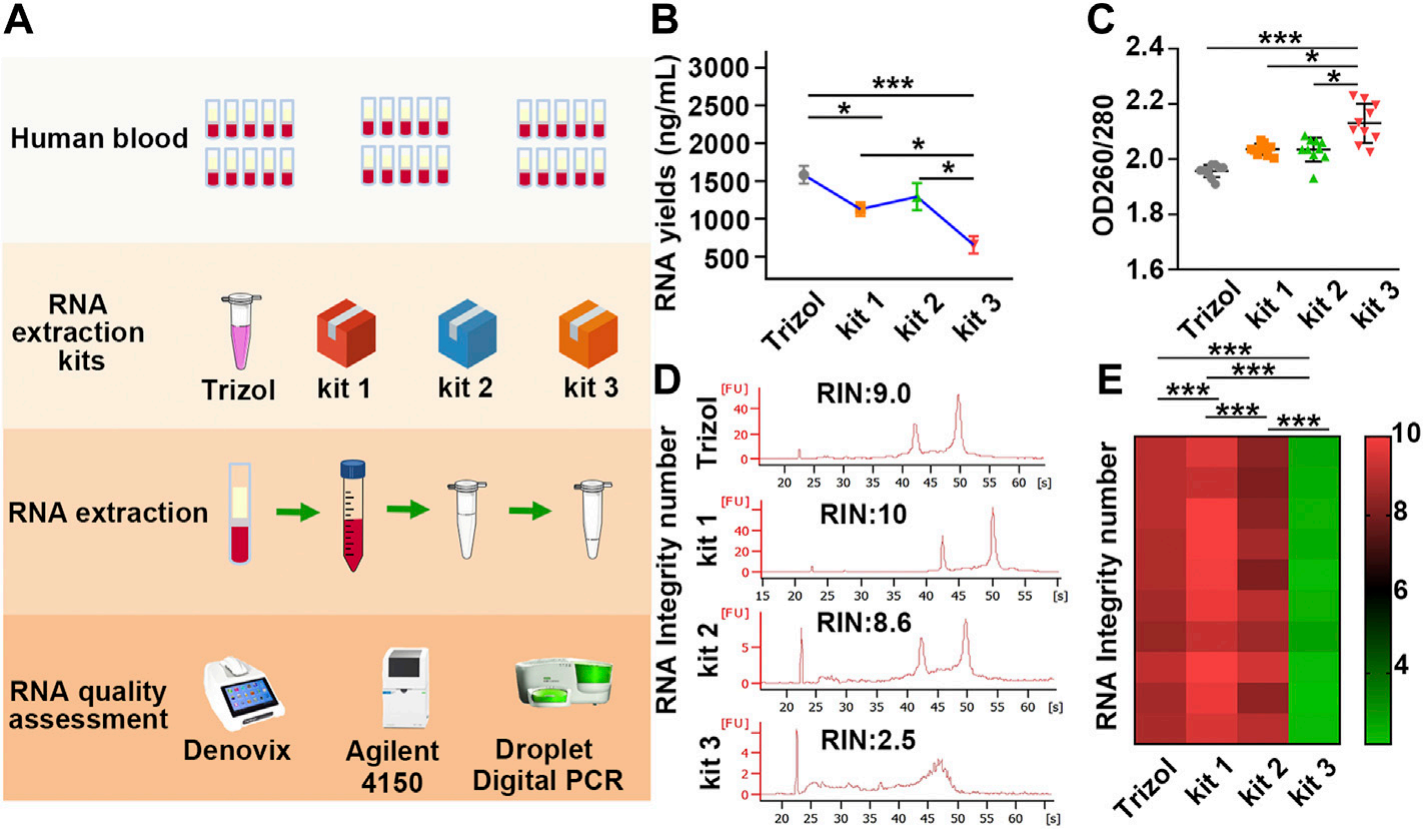
The yields, purity and RIN values of RNA extracted by different kits vary greatly. **(A)** Schematic illustration of the experimental schedule. **(B)** RNA yields in each group were assessed by Denovix. *n* = 40. **(C)** The 260/280 values were quantified using a DeNovix spectrophotometer. *n* = 40. **(D)** Representative image of RNA analysis by an Agilent bioanalyzer in each group. The first peak is a 20 bp molecular marker. The second and third peaks are 18 s and 28 s rRNA. **(E)** A heatmap based on RIN values in each sample. Red and green represent high and low RIN values, respectively. Data are represented as the mean ± SD (*n* = 10). Tamhane’s T2 test **(B,C)**, LSD test **(E)**, *p < .05, **p < .01, ***p < .001.

Various methods have been developed to purify PBL-derived RNA, and the red blood cell (RBC) lysis method has been primarily recommended for biobanks for further research (Heng et al., 2018). In recent years, several commercial kits have been successfully developed based on the RBC lysis method. However, due to the diversity of detection methods of various manufacturers, quality inconsistences may exist in RNA processed with different extraction kits. Therefore, it is necessary to evaluate the impact of different extraction kits on the quality of PBL-derived RNA. Thirty volunteers were recruited, and PBL-derived RNA was extracted immediately using three commercial RNA extraction kits: kit 1, kit 2, and kit 3. TRIzol reagent was employed as a standard control group to compare the quality and efficiency of different commercial kits. A novel enhanced evaluation system including RNA yields, purity, RIN values and β-actin copies was employed to identify the RNA quality differences (Figure 1A).

Our results showed that the yields of RNA extracted by TRIzol were slightly higher than those of RNA purified by kit 1 and kit 2, while the mean yields of RNA extracted by kit 3 were half as low as those of RNA purified by TRIzol, kit 1 and kit 2 (Figure 1B; Table 1). In addition, the 260/280 values of RNA extracted by kit 3 were considerably higher than 2 (Figure 1C; Table 1), suggesting the presence of protein contamination in RNA extracted by kit 3 (Manchester, 1996; Okamoto and Okabe, 2000; Wahlberg et al., 2012). The RIN value in each group was assessed using an Agilent 4150 Bioanalyzer, which can provide a separate RIN value as well as the correlating electrophoretic gel-like image for each sample (Imbeaud et al., 2005; Schroeder et al., 2006). As the results showed, RNA purified by TRIzol, kit 1 and kit 2 tended to be intact, and TRIzol and kit 1 exhibited better performance than kit 2. However, RNA extracted by kit 3 suffered severe degradation, with RIN values of only 2.5 ± 0.2 (Figures 1D, E; Supplementary Figure S1; Table 1). Gaps in RNA integrity may depend primarily on the composition of the three kits. In kit 1, β-mercaptoethanol (β-ME), a well-known reducing agent that irreversibly denatures RNase by reducing disulfide bonds and destroying the native conformation (van der Poel-van de Luytgaarde et al., 2013), was added to the leukocyte lysis buffer to eliminate the RNase. In kit 2, cells were lysed by guanidinium thiocyanate–phenol, which is also used in the TRIzol kit and can prevent the activity of RNA enzymes by denaturing them to yield undegraded RNA (Chomczynski and Sacchi, 1987). However, no RNase inactivator is labelled in Kit 3, which may be the main reason for the severe degradation of RNA. In support of this notion, we added 1% β-ME to the leukocyte lysis buffer. As our results showed, the presence of β-ME significantly increased the mean RIN value from 2.5 ± 0.2 to 7.0 ± 1.4 (Supplementary Figure S2; Supplementary Table S1), indicating that the poor performance of kit 3 was mainly due to the lack of RNase inactivator.

**TABLE 1:** The quality of PBL-derived RNA extracted by different kits varies widely^a^.

Kits	RNA yields (ng/ml)	OD260/280	RIN
TRIzol	1,580.25 ± 371.10	1.96 ± 0.02	8.9 ± 0.2
kit 1	1,125.37 ± 288.19	2.04 ± 0.02	9.7 ± 0.3
kit 2	1,290.80 ± 565.84	2.03 ± 0.04	8.6 ± 0.4
kit 3	652.43 ± 359.49	2.13 ± 0.07	2.5 ± 0.2

^a^ Results are means and standard deviations of ten independent extractions.

**FIGURE 2:**
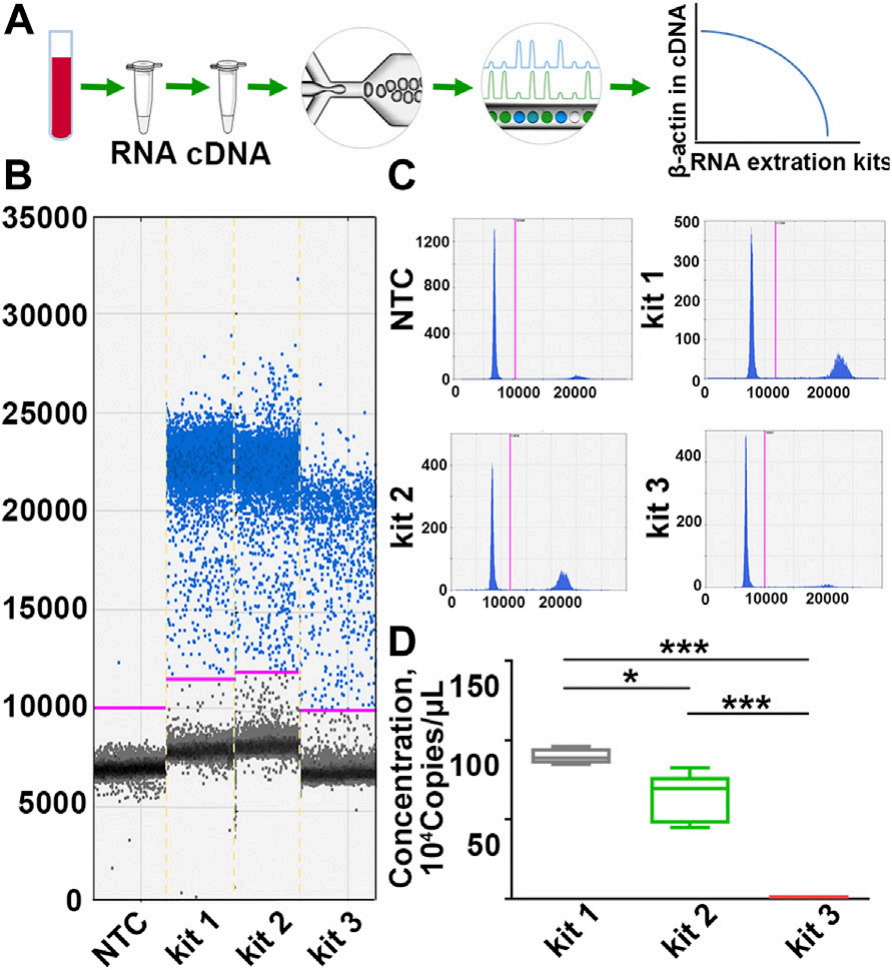
The copy number of β-actin is a more sensitive indicator to identify RNA quality inconsistencies. **(A)** A scheme for the detection principle of ddPCR. **(B,C)** Representative one-dimensional plots of droplets measured for fluorescence signal (ampli-tude indicated on *y*-axis) emitted from β-actin. Blue dots are positive signals, and black dots represent non-amplification signals. **(D)** Quantification of β-actin copies in samples processed with different RNA extraction kits. *n* = 30. Data are represented as the mean ± SD (*n* = 10). Tamhane’s T2 test (D), *p < .05, ***p < .001.

DdPCR was performed to detect the variance of β-actin copies to more accurately evaluate the inconsistencies of RNA in each group. In ddPCR, the reaction is divided into at least 10,000 partitions, and 40 cycles of classical PCR are carried out in each of these impervious nanocompartments. Those partitions with the amplified product are designated positive, and those with no amplified product are designated negative. Quantification was then achieved using Poisson statistics by counting fluorescence-positive and total droplet numbers (Passby et al., 2019) (Figure 2A). As the results showed, samples processed with kit 1 exhibited the highest β-actin copies. In particular, β-actin copies in samples processed with kit 1 or kit 2 were more than 400 times higher than those processed with kit 3 (Figures 2B–D; Supplementary Table S2), yet the variation in RIN values was only 4-fold across different groups, suggesting that the copy number of β-actin was more sensitive for identifying RNA integrity than RIN values. These results demonstrated that the quality of PBL-derived RNA extracted by different commercial kits varies widely, and β-actin copies can be a more sensitive indicator to assess RNA quality.

### 2.2 Blood placed at RT over 12 h significantly compromise PBL-derived RNA quality

Although the fresher the better is the golden rule when dealing with clinical samples, in most cases, blood collection and further RNA extraction are always conducted at different times and in different spaces, so it is necessary to find a balance between the storage conditions and RNA quality. Whereas previous studies have demonstrated that the storage temperature and duration are critical to RNA quality, the conclusion needs more verification due to the limited samples and evaluation system (Zhang et al., 2019).

**FIGURE 3:**
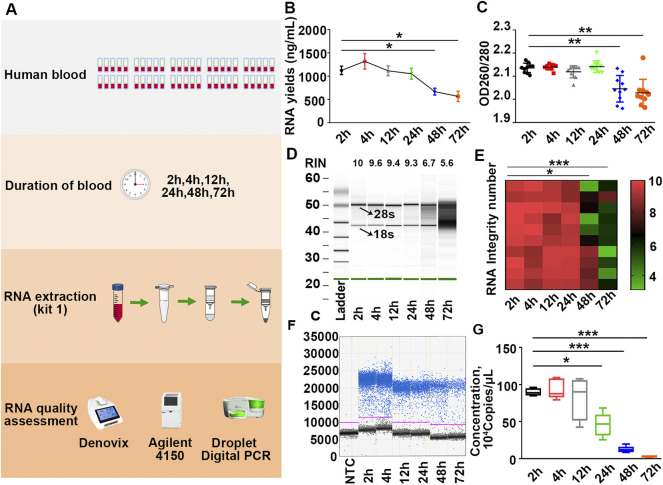
Placing blood at RT over 12 h significantly compromises RNA quality. **(A)** Schematic illustration of the experimental schedule. **(B,C)** Yields **(B)** and purity **(C)** of RNA in each group were measured by Denovix spectrophotometry. *n* = 60. **(D)** Representative electrophoretic gel-like image of RNA in each group. The arrows indicate 18 s and 28 s ribosomal bands. **(E)** Heatmap of RIN values of samples. *n* = 60. **(F)** Representative one-dimensional plots of droplets measured for fluorescence signal emitted from β-actin in each group. **(G)** Number of β-actin copies in each group. *n* = 60. Tamhane’s T2 test **(B,C,E,G)**, *p < .05, **p < .01, ***p < .001.

To explore the impact of the timing of blood placement at RT on the quality of PBL-derived RNA, sixty tubes of blood were placed on the lab bench at RT for 0, 30, 60, and 90 min before RNA extraction with kit 1 (Figure 3A). Our results showed that timing of blood at RT within 12 h displayed undetectable lesions on RNA quality. Prolonging the blood placement time to 24 h significantly compromised β-actin copies but had little effect on the yields, purity and RIN values of extracted RNA, confirming that the copy number of β-actin is a more sensitive indicator for RNA quality evaluation (Figures 3B–G; Supplementary Figure S3; Table 2). In particular, the longer the timing of blood placement, the poorer the RNA quality. Placing blood at RT for 48 h markedly compromised RNA yields, purity, RIN values and β-actin copies (Figures 3B–G; Supplementary Figure S3; Table 2). These results implied that the timing of blood placement at RT should be performed within 12 h to obtain desirable RNA.

**TABLE 2:** Blood placed at RT over 12 h significantly compromised PBL-derived RNA quality^a^.

Time(h)	RNA yields (ng/ml)	OD260/280	RIN	β-actin copies (Copies/μl)
2	1,125.37 ± 288.19	2.04 ± 0.02	9.7 ± 0.3	897,200 ± 51,621
4	1,319.51 ± 524.09	2.04 ± 0.01	9.4 ± 0.5	938,560 ± 135,087
12	1,116.02 ± 336.48	2.02 ± 0.03	9.4 ± 0.4	826,400 ± 294,752
24	1,053.13 ± 372.91	2.04 ± 0.03	9.4 ± 0.4	457,280 ± 159,569
48	670.14 ± 231.77	1.95 ± 0.06	6.6 ± 2.0	123,872 ± 45,041
72	567.16 ± 352.47	1.93 ± 0.06	5.5 ± 1.3	29,600 ± 6,556

^a^ Results are means and standard deviations of ten independent extractions.

### 2.3 Compromised RNA leads to skewed mRNA and lncRNA readouts in patients with COPD or TNBC

**FIGURE 4:**
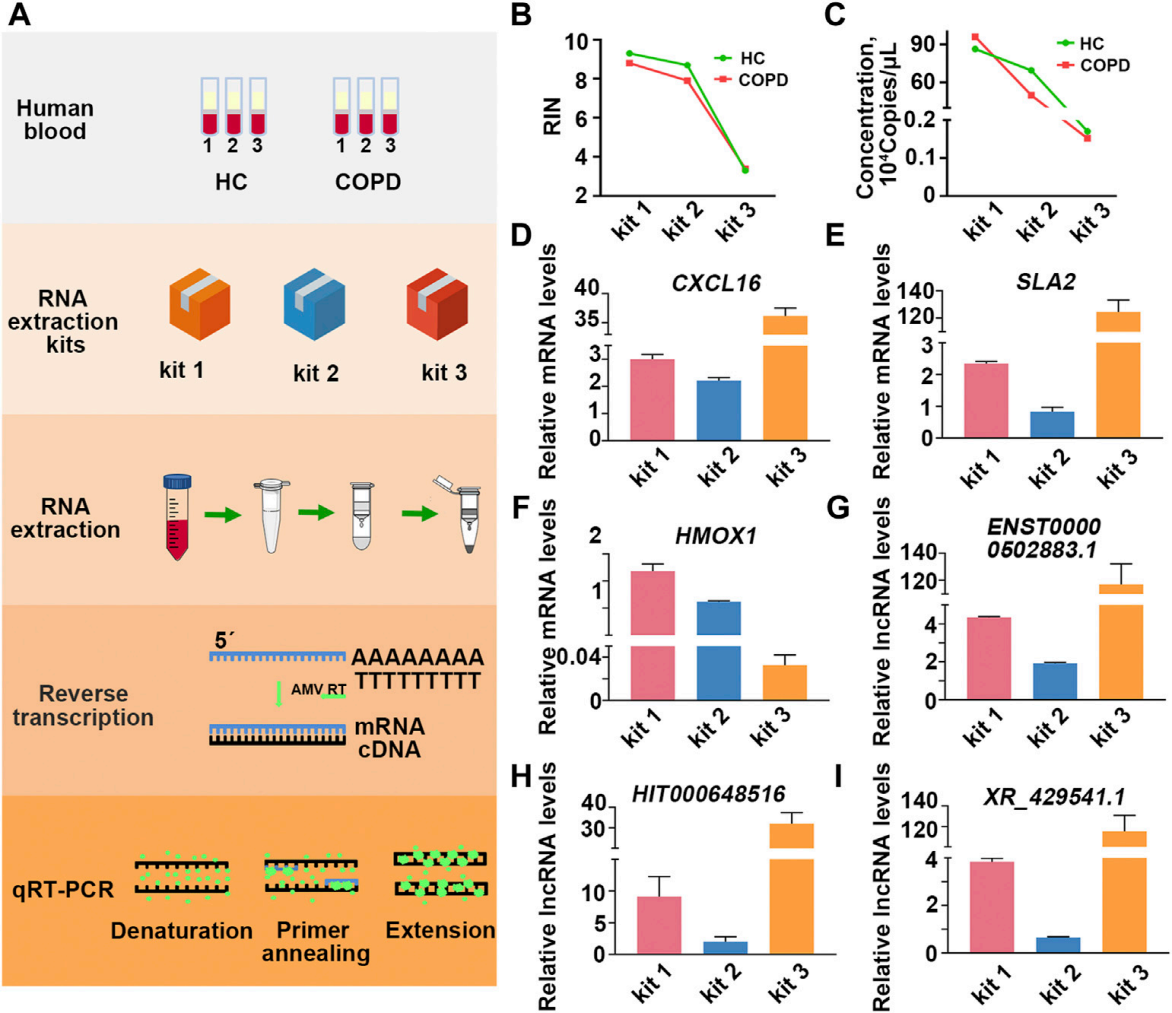
Comprised RNA leads to skewed transcriptional readouts in COPD patient. **(A)** Schematic illustration of the experimental schedule. **(B)** The RIN values in each group. **(C)** The quantification of β-actin copies in each group. **(D–F)** The relative transcriptional readouts of the informative mRNAs in COPD patient in each group. **(G–I)** The relative informative lncRNAs readouts in COPD patient in each group.

Having found the influence of extraction kits and the timing of blood placement on PBL-derived RNA quality, we further investigated the impact of RNA quality on downstream transcriptional readouts. Inflammation plays a pivotal role in the pathogenesis of COPD, where CD8^+^ T lymphocytes, neutrophils, and macrophages are the main types of immune cells of the local inflammatory milieu (Cosio et al., 2002; Stankiewicz et al., 2002). Previous studies have identified informative mRNAs (CXCL16, HMOX1, SLA2, *etc.*) and lncRNAs (ENST00000502883.1, HIT000648516, XR_429541.1, *etc.*) in peripheral blood mononuclear cells (PBMCs) from COPD patients *versus* smokers (Sui et al., 2013; Song et al., 2015; Dang et al., 2017; Qu et al., 2018). Here, a smoker and a COPD patient were recruited, and the blood of each individual was equally divided into three aliquots for RNA isolation by the three RNA extraction kits. The expression of informative mRNAs (CXCL16, HMOX1, SLA2) and lncRNAs (ENST00000502883.1, HIT000648516, XR_429541.1) was detected in each sample (Figure 4A).

As the results showed, the quality of RNA extracted by different kits varied greatly even for the same individual (Figures 4B,C; Supplementary Figure S4; Supplementary Table S3). To minimize the interference of RNA inputs on transcriptional readouts, we performed relative quantification according to the ΔΔCt method, and the expression level of β-actin was used as an internal control (Nolan et al., 2006). As the results showed, when PBL-derived RNA was extracted by kit 1, the mean relative transcriptional readout of CXCL16 in COPD patient was 3 times higher than that in healthy control (HC) (Figure 4D). However, the alteration of CXCL16 mRNA levels between the two individuals was approximately 2-fold and 40-fold when samples were processed with kit 2 and kit 3, respectively (Figure 4D; Table 3). Similar results were observed in another informative COPD marker, SLA2 (Figure 4E; Table 3). Even more striking, the relative transcriptional readout of HMOX1 in COPD patient was markedly increased in RNA extracted by kit 1 but significantly decreased in RNA treated with kit 3 (Figure 4F; Table 3). Moreover, the relative transcriptional readouts of informative lncRNAs in COPD (ENST00000502883.1, HIT000648516, XR_429541.1) were also affected by PBL-derived RNA quality, and compromised RNA significantly resulted in skewed results (Figures 4G–I; Table 3).

**TABLE 3:** The relative informative mRNA and lncRNA readouts in COPD in each group.

Kits/RNA	*CXCL16*	*HMOX1*	*SLA2*	*ENST00000*	*HIT000*	*XR_429541.1*
				*502,883.1*	*648,516*	
kit 1	3.00 ± 0.14	1.69 ± 0.10	2.35 ± 0.05	4.34 ± 0.04	9.13 ± 2.56	3.83 ± 0.12
kit 2	2.22 ± 0.09	1.11 ± 0.02	0.83 ± 0.11	1.91 ± 0.04	2.02 ± 0.63	0.65 ± 0.03
kit 3	36.20 ± 1.10	0.03 ± 0.01	124.60 ± 7.11	117.15 ± 12.31	32.11 ± 4.34	135.17 ± 12.87

In addition to inflammatory disease, interactions between the immune system and tumors are highly reciprocal in nature, and the presence of cancer cells causes immune cells to undergo various phenotypic and functional changes (Dirkx et al., 2006; Talmadge and Gabrilovich, 2013; Noy and Pollard, 2014; Suzuki et al., 2019). Based on these concepts, attempts have been made to detect the presence of cancer cells by analyzing the gene expression profile of PBMCs in patients with lung cancer, pancreatic cancer and breast cancer (Saeed et al., 2003; Showe et al., 2009). TNBC is a very aggressive subtype of normal breast cancer, and great efforts have been made to identify informative mRNAs and lncRNAs in TNBC. A previous study reported that TLR4, TNF receptor-associated factor 6 (TRAF6), and TGF-beta receptor type I (TGFβRI) were considerably upregulated in TNBC patients. In addition, lncRNA-ATB (Li et al., 2018), metastasis-associated lung adenocarcinoma transcript 1 (MALAT1) and lncRNA H19 (Jin et al., 2016; Li et al., 2020; Huang et al., 2021), which are abnormally expressed in TNBC, may provide a less invasive diagnostic procedure to reveal immunological insight of breast cancer. Thus, we explored the influence of PBL-derived RNA quality on the transcriptional readouts of informative mRNAs and lncRNAs in TNBC.

**FIGURE 5:**
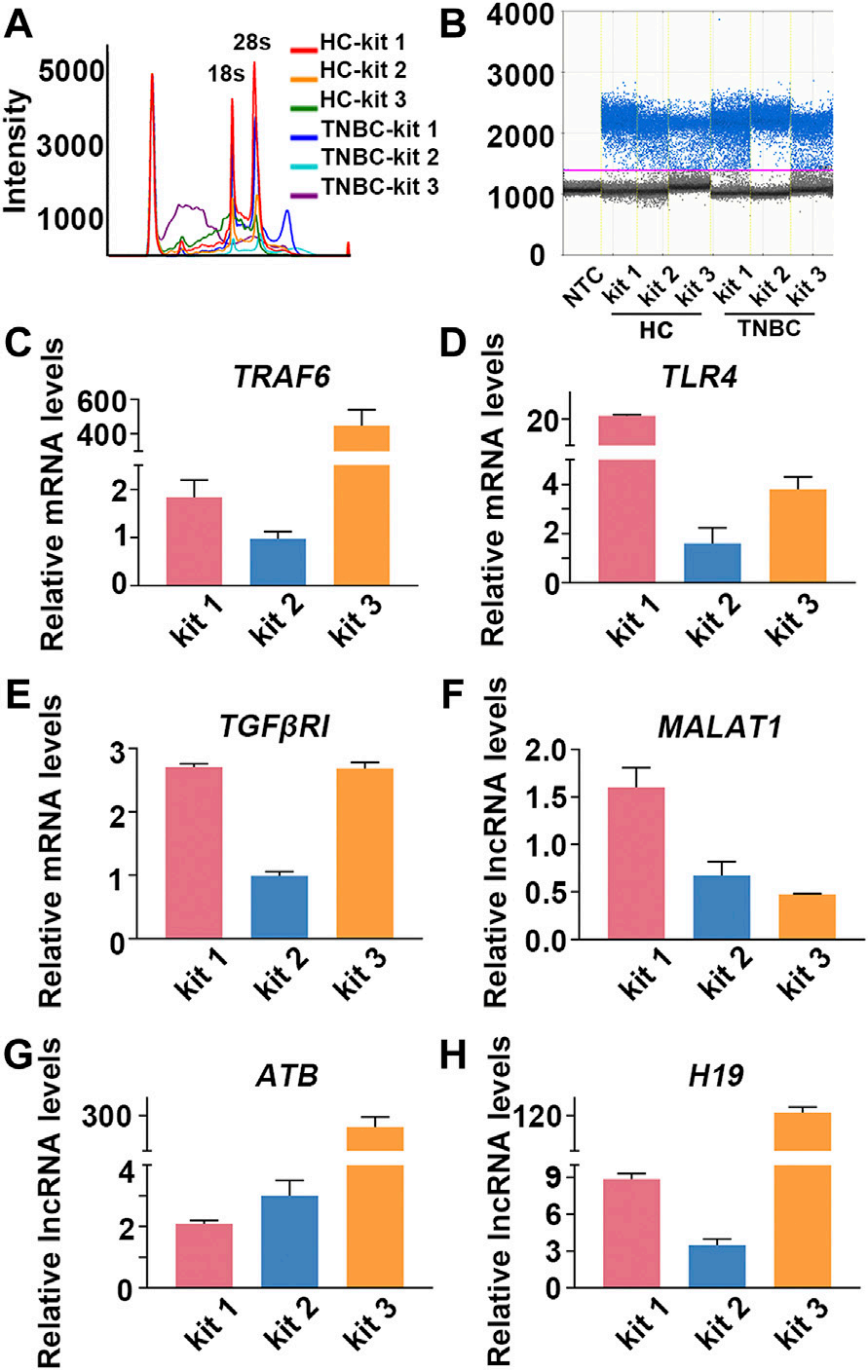
Compromised RNA results in skewed mRNA and lncRNA readouts in TNBC patient. **(A)** Representative image of RNA analysis by an Agilent bioanalyzer in each group. **(B)** Representative one-dimensional plots of droplets measured for fluorescence signal emitted from β-actin in each group. **(C–H)** The relative transcriptional readouts of the informative mRNAs and lncRNAs in TNBC patient in each group.

As the results showed, the quality of RNA extracted by different kits was quite different in each individual. The performance of kit 1 was better than that of kit 2, and RNA extracted by kit 3 suffered severe degradation (Figures 5A,B; Supplementary Figure S5; Supplementary Table S4). Importantly, the relative transcriptional readouts of TRAF6 in TNBC patient were 1.8 times higher than that detected in HC when RNA was extracted by kit 1 (Figure 5C; Table 4). However, there was no difference in the expression level of TRAF6 when samples were treated with kit 2, and processing samples with kit 3 resulted in a suspicious increase in the mRNA content of TRAF6 (Figure 5C; Table 4). The expression level of TLR4 in TNBC patient was 21-fold higher than that in HC when RNA was isolated by kit 1, while a significant decrease in TLR4 expression in patient were observed when samples were processed with kit 2 and kit 3 (Figure 5D; Table 4). Interestingly, for TGFβRI readouts, there was no difference between the samples processed with kit 1 and kit 3 (Figure 5E; Table 4). In accordance with the mRNA results, the relative transcriptional readouts of the same lncRNA in each sample processed with distinct extraction kits were quite different and irregular (Figures 5F–H; Table 4). These results demonstrated that the transcriptional readouts of mRNAs and LncRNAs were heavily dependent on the quality of extracted RNA, and improper preanalytical handling may lead to skewed results.

**TABLE 4:** The relative transcriptional readouts of informative mRNAs and lncRNAs in TNBC in each group.

Kits/RNA	*TLR4*	*TGFβ1*	*TRAF6*	*MALAT-1*	*ATB*	*H19*
kit 1	21.25 ± 0.34	2.71 ± 0.04	1.84 ± 0.29	1.60 ± 0.17	2.09 ± 0.09	8.86 ± 0.37
kit 2	1.59 ± 0.52	0.99 ± 0.05	0.97 ± 0.12	0.67 ± 0.12	3.00 ± 0.41	3.48 ± 0.41
kit 3	3.80 ± 0.41	2.69 ± 0.08	449.18 ± 74.91	0.48 ± 0.01	268.23 ± 23.23	121.88 ± 2.51

## 3 Conclusion and discussion

Genetic and epigenetic reprogramming caused by disease states in other tissues are systemically reflected in peripheral blood leukocytes (Javierre et al., 2010; Wang et al., 2010; Smith et al., 2014). As the most important fraction of leukocytes, RNA has been commonly used to study the response of transcriptome to disease-related stress. A large number of studies have demonstrated that mRNAs and lncRNAs are involved in various diseases, such as inflammation and malignancy, and aberrantly expressed mRNAs and lncRNAs are considered possible strong biomarkers. An accurate transcriptional readout of mRNA and lncRNA is fundamental for disease-related study, diagnosis and treatment. However, studies of preanalytical variables on RNA quality and downstream mRNA and lncRNA readouts are still lacking.

Numerous studies have demonstrated that the process of PBL-derived RNA extraction is susceptible to the variability of many factors (De Cecco et al., 2009; Rudloff et al., 2010; Hatzis et al., 2011), while the potential impact of different extraction kits on extracted RNA quality remains poorly understood. In addition, although some experiments suggested that storage time was critical for RNA quality (Zhang et al., 2019), the impact of timing of blood placement at RT on RNA quality needs more verification due to the limited samples and evaluation system. Moreover, some reports have pointed out that conventional indicators, such as RNA yields, purity and RIN values, might not be sensitive enough to discern between low- and high-quality RNA (Ribeiro-Silva et al., 2007; Greytak et al., 2015; Webster et al., 2015; Hester et al., 2016), suggesting that an enhanced evaluation system should be constructed to assess RNA quality inconsistencies.

qRT–PCR is the most commonly used technique to detect the expression of mRNA and lncRNA. Some researchers have explored the correlation between RIN values and qRT–PCR results and found that the expression values of 4 housekeeping genes (GAPDH, KYNF, NEFL, β2M) were heavily reliant on RNA integrity (Schroeder et al., 2006). Additionally, Ct values had an opposite trend compared to the RIN (Wang et al., 2015). A few studies have assessed the influence of RNA integrity on CP and delta CP. Their findings showed that CP decreased with increasing RIN, while delta CP was slightly affected (Fleige and Pfaffl, 2006). However, little is known about the effect of RNA quality on relative gene expression calculated by delta-delta Ct, which has been the most preferred method for qRT–PCR data analysis.

In this study, we first assessed the effects of extraction kits and timing of blood placement on PBL-derived RNA quality. A novel enhanced evaluation system including RNA yields, purity, RIN values and β-actin copies was employed to identify RNA differences. We found that the quality of RNA extracted by kit 1 and kit 2 was comparable to that of RNA purified by TRIzol, and kit 1 had a better performance than kit 2 in RIN values and β-actin copies. However, RNA extracted by kit 3 was subjected to severe degradation due to the lack of RNase inactivator, suggesting that kit evaluation and management should be performed before RNA extraction in batches. In addition, placement of blood at RT over 12 h significantly compromised the copy number of β-actin, indicating that the timing of blood placement at RT should be within 12 h to obtain desirable RNA. More importantly, the relative transcriptional readouts of the informative mRNAs and lncRNAs in patients with TNBC or COPD were heavily dependent on the quality of extracted RNA, and improper preanalytical handling may lead to skewed results. Our findings thus exhibited significant implications for PBL-derived RNA quality assessment and downstream qRT–PCR analysis.

## 4 Materials and methods

### 4.1 Blood collection

In brief, 5 ml blood in each sample was centrifuged at 1,500 g for 10 min at 4°C. After centrifugation, three different fractions are distinguishable: The upper layer is plasma; the intermediate layer is buffy coat, which concentrates leukocytes; and the bottom layer contains concentrated erythrocytes. The buffy coat was then harvested, and RBC lysis buffer was added to lyse the remaining erythrocytes. After centrifugation, total WBCs were harvested from the pellets. The following procedure was carried out according to the manufacturer’s instructions for the evaluated RNA extraction kits (kit 1, kit 2 and kit 3).

A COPD patient was eligible for this study if he met the following criteria: smoking history (≥20 pack years); postbronchodilator FEV1 ≥ 25% of the predicted value and postbronchodilator FEV1/forced vital capacity (FVC) ≤ 0.70; and no history of asthma, atopy (as assessed by an allergy skin prick test during screening) or any other active lung disease.

A TNBC patient was eligible for this study if she met the following criteria: grade 2 or 3 infiltrating ductal carcinoma with negative expression of ER, PR and HER2 proteins or accompanied by medullary features, infiltrating micropapillary carcinoma, or occasional vascularized thrombus.

### 4.2 RNA quantification

The yields and purity of extracted RNA were assessed by using a denovix spectrophotometer. The absorption at UV 260 nm was used to assess the RNA yields, and the ratio of 260 nm and 280 nm was used to evaluate RNA purity. An Agilent 4150 Bioanalyzer and the RNA 6000 Nano LabChip kit were employed to calculate the RNA Integrity Number (RIN). The RIN index ranges from 1 to 10, with 1 indicating the greatest degradation and 10 being the most intact RNA (Schroeder et al., 2006). The copy number of β-actin was detected on a digital droplet PCR (ddPCR) platform. Briefly, RNA in each group was reverse-transcribed into cDNA with the ReverTraAce qRT–PCR RT Kit (TOYOBO, Cat: FSQ-101). The aqueous ddPCR mixture containing 10 µl of ddPCR™ EvaGreen Supermix (BIO-RAD, Cat: #1864033), 3 µl of β-actin primers (3.75 µM) and 7 µl of cDNA was emulsified into picoliter droplets of thermostable oil in a QX200™ Droplet Generator. Subsequently, β-actin was amplified on a QX200 PCR system (Bio-Rad) at 95°C (30 s) and 60°C (60 s) for 40 PCR cycles. The ramp rate between any two consecutive steps was set to 2°C to ensure reliable thermal control. Next, the positive *versus* negative droplets were read by a QX200™ Droplet Reader, and the absolute quantification of β-actin was calculated using QuantaSoft software (Bio-Rad). Primer sequences for ddPCR are listed in Supplementary Table S5.

### 4.3 Real-time PCR

The relative transcriptional readouts of the informative mRNAs and LncRNAs in patients with COPD or TNBC were measured by qRT–PCR. Quantitative PCR was performed with SYBR Green Real-Time PCR Master Mix (TOYOBO, Cat: QPK-201) in the Real-Time PCR System (Bio-Rad, CFX96). The cycling profile was as follows: initial denaturation at 95°C for 5°min, followed by 40 cycles with 20 s at 95°C, 20 s at 60°C and 30 s at 72°C. The expression level of β-actin was used as an internal control. Primer sequences for qRT–PCR are listed in Supplementary Table S5.

### 4.4 Statistical analysis

Statistical analysis was performed using SPSS version 21 (IBM Corp.). All statistical graphs were constructed using Prism 8.0 (GraphPad Software, Inc.). All the results are presented as the mean ± standard deviation (SD). The comparison between groups was drawn through *t*-test and analysis of variance. Multiple comparison correction was conducted by Tamhane’s T2 (or T2’) and LSD(L) test for normally distributed data with unequal or equal variances, respectively. *P* < .05 was considered to indicate a statistically significant difference.

## Data Availability

The original contributions presented in the study are included in the article/Supplementary Material, further inquiries can be directed to the corresponding authors.
