# Supplementary material for: Improper preanalytical processes on peripheral blood compromise RNA quality and skew the transcriptional readouts of mRNA and LncRNA
Source: Front Genet. 2023 Jan 4;13:1091685. doi: 10.3389/fgene.2022.1091685 (PMC9845260; doi:10.3389/fgene.2022.1091685)
Supplement: Supplementary file 1 [file DataSheet1.DOCX]

Supplementary Material

# Supplementary Figures and Tables

## Supplementary Figures


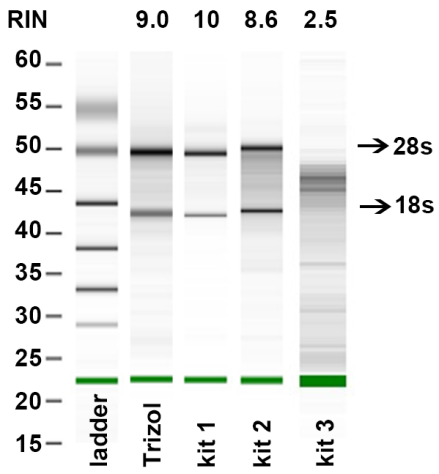


**Supplementary Figure 1.** Representative electrophoretic gel-like image of RNA in each group. The arrows indicate 18s and 28s ribosomal bands.


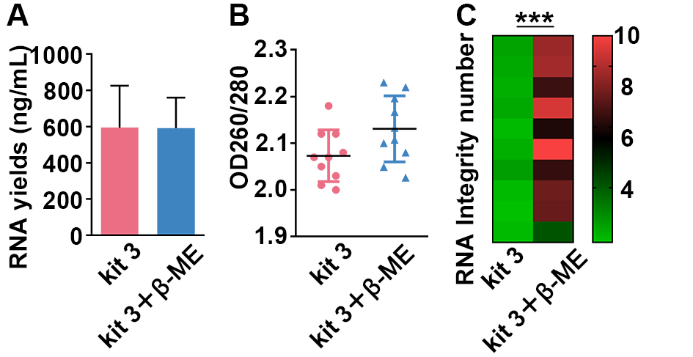


**Supplementary Figure 2.** The presence of β-ME rescues the poor performance of kit 3. (A-C) The yields (A), purity (B) and RIN values (C) of RNA in the absence or presence of β-ME. Data are represented as the mean ± SD (n=10). *t* test (C), *** *p*<0.001.


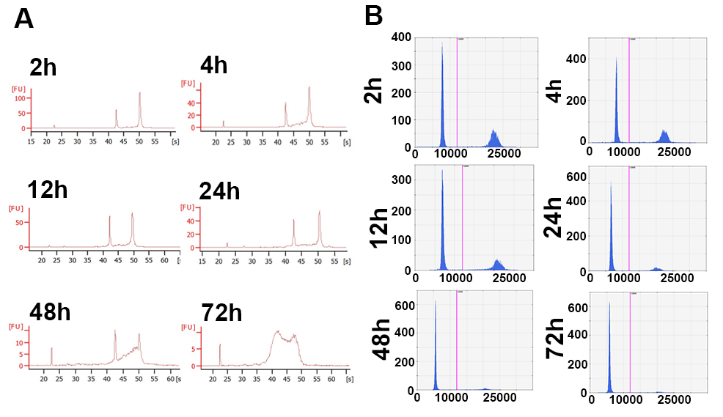


**Supplementary Figure 3.** The integrity of RNA in each group was detected using the Agilent Bioanalyzer 2100 and ddPCR. (A) Densitometry traces showed that preserved blood at RT within 24 hours displayed undetectable lesions on RNA quality. (B) Histograms of ddPCR showed that preserved blood at RT within 12 hours displayed undetectable lesions on RNA quality.


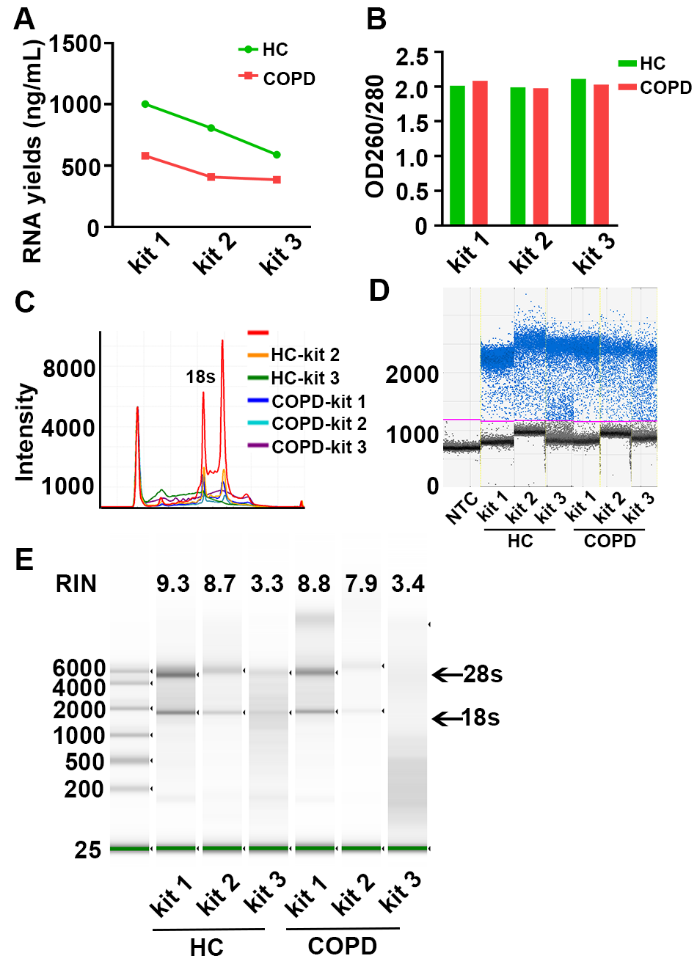


**Supplementary Figure 4.** The quality of RNA purified by different kits in healthy control and COPD patient. (A and B) Yields (A) and purity (B) of RNA in each group were measured by Denovix spectrophotometry. (C) Representative image of RNA analysis by an Agilent bioanalyzer in each group. (D) Representative one-dimensional plots of droplets measured for fluorescence signal emitted from β-actin in each group. (E) Representative electrophoretic gel-like image of RNA in each group. The arrows indicate 18s and 28s ribosomal bands.


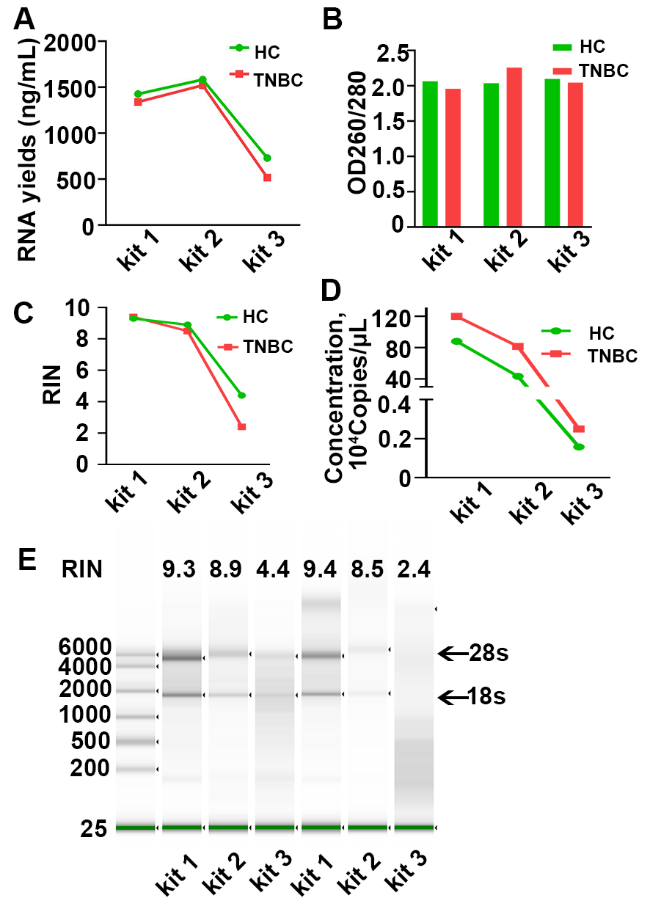


**Supplementary Figure 5.** The quality of RNA purified by three evaluated kits in healthy control and TNBC patient. (A and B) Yields (A) and purity (B) of RNA in each group. (C-D) The integrity of RNA and β-actin copies were detected by an Agilent Bioanalyzer 2100 and ddPCR, respectively. (E) Representative electrophoretic gel-like image of RNA in each group. The arrows indicate 18s and 28s ribosomal bands.

## Supplementary Tables

# Supplementary Table 1. The yields, purity and RIN values of RNA extracted by Kit 3 in the absence or presence of β-ME.

| **Kits** | **RNA yields**  **(ng/mL)** | **OD260/280** | **RIN** |
| --- | --- | --- | --- |
| kit 3 | 652.43±359.49 | 2.13±0.07 | 2.5±0.2 |
| kit 3+β-ME | 592.42±167.84 | 2.07±0.06 | 7.0±1.4 |

# Supplementary Table 2. Beta-actin copies in samples processed with different RNA extraction kits.

| **Kits** | **β-actin copies (Copies/μL)** |
| --- | --- |
| kit 1 | 897200±51621 |
| kit 2 | 637440±157738 |
| kit 3 | 1572±116 |

# Supplementary Table 3. The quality of RNA purified by different kits in healthy control and COPD patient.

| **Kits** | **Healthy control** | | | |  | **COPD patient** | | | |
| --- | --- | --- | --- | --- | --- | --- | --- | --- | --- |
|  | **RNA yields**  **(ng/mL)** | **OD260**  **/280** | **RIN** | **β-actin copies**  **(Copies/μL)** |  | **RNA yields**  **(ng/mL)** | **OD260**  **/280** | **RIN** | **β-actin copies**  **(Copies/μL)** |
| kit 1 | 1001.584 | 2.01 | 9.3 | 864000 |  | 580.616 | 2.08 | 8.8 | 961600 |
| kit 2 | 806.752 | 1.99 | 8.7 | 696000 |  | 408.272 | 1.98 | 7.9 | 499200 |
| kit 3 | 589.04 | 2.11 | 3.3 | 1704 |  | 385.024 | 2.03 | 3.4 | 1520 |

# Supplementary Table 4. The quality of RNA purified by three evaluated kits in healthy control and TNBC patient.

| **Kits** | **Healthy control** | | | |  | **TNBC patient** | | | |
| --- | --- | --- | --- | --- | --- | --- | --- | --- | --- |
|  | **RNA yields**  **(ng/mL)** | **OD260**  **/280** | **RIN** | **β-actin copies**  **(Copies/μL)** |  | **RNA yields**  **(ng/mL)** | **OD260**  **/280** | **RIN** | **β-actin copies**  **(Copies/μL)** |
| kit 1 | 1427.336 | 2.06 | 9.3 | 881600 |  | 1341.312 | 1.96 | 9.4 | 1200000 |
| kit 2 | 1584.816 | 2.04 | 8.9 | 433600 |  | 1523.056 | 2.26 | 8.5 | 816000 |
| kit 3 | 731.04 | 2.10 | 4.4 | 1576 |  | 517.816 | 2.05 | 2.4 | 2488 |

# Supplementary Table 5. Primer sequences for ddPCR and qRT-PCR

| **Gene name** | **Primers** |
| --- | --- |
| *β-actin* | F: CATGTACGTTGCTATCCAGGC |
|  | R: CTCCTTAATGTCACGCACGAT |
| *TLR4* | F: GGCATTTAGGCAGCTATAGCTTC |
|  | R: TTAAGGTAGAGAGGTGGCTTAGGC |
| *TGFβRI* | F: GTCTGTGACTACAACATATTGCTGC |
|  | R: GAAGCACACTGGTCCAGCAATG |
| *TRAF6* | F: ATTACGAGAAGCAGTGCAAACG |
|  | R: CTCACGTTTTGCAAAATTGTCTGG |
| *MALAT1* | F: GATTGAGGAGGCTGTGCTGT |
|  | R: CAGCTGCCTGCTGTTTTCTG |
| *ATB* | F: CTTCACCAGCACCCAGAGA |
|  | R: AAGACAGAAA AACAGTTCCG AGTC |
| *H19* | F: AGCGGGTCTGTTTCTTTACTT |
|  | R: AGCTGGGTAGCACCATTTC |
| *CXCL16* | F: CTGAGAGCTTACCATCGGTGT |
|  | R: TCAAGACAGCTCATCAATTCCT |
| *HMOX1* | F: GGCCAGCAACAAAGTGCAAG |
|  | R: ATGGCATAAAGCCCTACAGCA |
| *SLA2* | F: GACATCTGCTGCCTACTCAAGG |
|  | R: TGTGGCAGCTTCAGAAAACAGG |
| *ENST00000502883.1* | F: GTGTCCATGTAACTAACTCCTGGT |
|  | R: GTCAGCGGGAAGGAAGACAG |
| *HIT000648516* | F: ATAGAAGCGATCTACCCTCACAG |
|  | R: TGCTGGGCTCGTTCGT |
| *XR_429541.1* | F: TGGTCACTTTCCAGTTTCCACA |
|  | R: AATTCAGATCCCACATCAGCCT |

# F: forward; R: reverse.
